# Polarization perception in humans: on the origin of and relationship between Maxwell’s spot and Haidinger’s brushes

**DOI:** 10.1038/s41598-019-56916-8

**Published:** 2020-01-10

**Authors:** Gary P. Misson, Shelby E. Temple, Stephen J. Anderson

**Affiliations:** 1 School of Life & Health Sciences, Aston University, Birmingham, B4 7ET UK; 2 Department of Ophthalmology, South Warwickshire NHS Foundation Trust, Lakin Road, Warwick, CV34 5BW UK; 3 School of Biological Sciences, University of Bristol, Bristol, BS8 1TQ UK; 4 Azul Optics Ltd, Bristol, BS10 5BD UK; 5 School of Life & Health Sciences, Aston University, Birmingham, B4 7ET UK

**Keywords:** Computational biophysics, Retina

## Abstract

Under specific conditions of illumination and polarization, differential absorption of light by macular pigments is perceived as the entoptic phenomena of Maxwell’s spot (MS) or Haidinger’s brushes (HB). To simulate MS and HB, an existing computational model of polarization-dependent properties of the human macula was extended by incorporating neuronal adaptation to stabilized retinal images. The model predicted that polarized light modifies the appearance of MS leading to the perception of a novel phenomenon. The model also predicted a correlation between the observed diameters of MS and HB. Predictions were tested psychophysically in human observers, whose measured differences in the diameters of each entoptic phenomenon generated with depolarized and linearly polarized light were consistent with the model simulations. These findings support a common origin of each phenomenon, and are relevant to the clinical use of polarization stimuli in detecting and monitoring human eye disorders, including macular degeneration. We conclude: (i) MS and HB both result from differential light absorption through a radial diattenuator, compatible with the arrangement of macular pigments in Henle fibres; (ii) the morphology of MS is dependent on the degree of linear polarization; (iii) perceptual differences between MS and HB result from different states of neural adaptation.

## Introduction

The human macula is the retinal area anatomically and functionally optimised for high visual acuity. Centred on the visual axis of the eye, it derives its name (*macula lutea* = yellow spot) from its high concentration of plant-derived xanthophyll carotenoid pigments (i.e. macular pigments). Macular pigments are thought to protect against the damaging effects of high-energy visible wavelengths (380–500 nm) by acting as violet/blue light filters and free-radical scavengers^1–3^. Additional vision-related functions include the reduction of chromatic aberration^4^ and light scatter^5^. It is generally thought that low concentrations of macular pigment predispose to eye disease, particularly age-related macular degeneration^6–9^.

The high concentrations of yellow pigments within the human central macula result in a reduction of up to 80% of high-energy visible light reaching the photoreceptor outer segments^10^. Nonetheless, under normal viewing conditions, variations in pigment concentration across the macula do not manifest as perceptual differences in colour and/or luminance. This is because adaptive mechanisms function to negate the perception of unchanging retinal images (e.g. the Troxler effect)^11,12^.

Inhomogeneities in retinal anatomy and function become visible when adaptive mechanisms are disrupted, such as in the case of Maxwell’s spot (MS)^13^. This entoptic phenomenon results from a change in illumination of macular photoreceptors achieved, for example, by alternately viewing uniformly illuminated fields of visible light that are either absorbed (wavelengths 380–520 nm, blue) or transmitted (adapting wavelengths 520–700 nm, e.g. yellow) by macular pigment. Under these conditions MS appears as a dark circular spot subtending 2°–3° in angular diameter, centred at the point of fixation. The appearance of MS is dependent on viewing conditions (e.g. wavelengths of the viewing fields and their temporal modulation), with considerable inter-observer variability in its appearance for any given method of observation^11,14^. Distinction is made here between MS and the S-cone scotoma. The latter arises from the absence of S-cones within the foveola and, although often elicited coincidentally with MS, it has a much smaller diameter (20–30′) and is unrelated to macular pigment^15,16^.

Maxwell linked MS to another entoptic phenomenon described twelve years earlier by Haidinger^17^, later given the name Haidinger’s brushes (HB). Observing a uniform field of linear polarized light, Haidinger noted a faint yellowish hour-glass like percept confined within the central 3° of fixation. This phenomenon rapidly fades due to neural adaptation. It can be made to persist by refreshing the retinal image by rotating the orientation of incident linear polarization^18^. Following a series of insightful experiments, Maxwell^19^ proposed that HB was generated by selective absorption of polarized light by a radially symmetric diattenuating retinal structure centred on the fovea. This hypothesis has received considerable experimental support, and it is now generally accepted that HB is due to the presence of dichroic pigment molecules within radially symmetric macular structures, principally macular pigments within the photoreceptor axons comprising the Henle fibre layer^18^. Apart from relating both HB and MS to macular pigment (‘*the yellow spot on the retina*’), Maxwell went on to conjecture that HB was MS ‘*analysed by polarized light*’^13^. Maxwell’s conjecture implies a common mechanism for both phenomena, but does not explain their morphological differences or reasons for their different modes of generation.

In this study, we aim to clarify the relationship between MS and HB and the role of neural adaptive mechanisms in their genesis. We firstly used an established computational model based on the known radial diattenuating properties of the central macula to determine the spatial variation of light intensity reaching the array of photoreceptor outer segments for both depolarized and linearly polarized light. We next considered the effect of neural adaptation by subtracting, from the total photoreceptor illumination, those components that remain constant over time (i.e. behave as a spatially and temporally stabilised image). The predictions of the model were tested *in vivo* in human participants by determining whether the observed diameter of MS is polarization-dependent and whether it is correlated with the maximum observed diameter of HB.

Our theoretical and experimental results allow a unifying model of MS and HB, and provide novel insight into the role of neural adaptation. This is important because of the possible clinical application of these phenomena in the diagnosis and monitoring of various eye and vision-related disorders^20–22^, including amblyopia^23^, dyslexia^24^ and age-related macular degeneration^25^. Quantification of HB perception has recently been proposed as a rapid and easy method for assessing macular pigment density in otherwise healthy individuals as part of health screening and blue-light hazard avoidance^26^. Advancing our understanding of these phenomena, in terms of both their generation and relationship, is essential for their full utilisation as diagnostic and investigative ophthalmic tools.

## Theoretical

### Methods

Our computational model is derived from that of Misson *et al*.^27,28^, in which incident polarized light interacts with a radial diattenuator (an appropriate model for the macular pigments bound in the Henle fibre layer) defined by maximum (*k*_1_) and minimum (*k*_2_) principal transmittances. In this study, emphasis is given to the effect of the degree of polarization (*P*) of incident light and the values of *k*_1_ and *k*_2_ on light transmission through the radial diattenuator, thereby simulating intensity of light reaching the photoreceptor layer of the retina for incident light of a given degree of polarization and **E**-vector orientation.

The 2-dimensional Stokes-Mueller formulation of the system is:

1 $${{\bf{S}}}_{{\bf{o}}{\bf{u}}{\bf{t}}}[\theta ,{k}_{1},{k}_{2},P,{\epsilon }]={{\bf{M}}}_{{\bf{M}}}[\theta ,{k}_{1},{k}_{2}]{{\bf{M}}}_{{\bf{D}}}[P]{{\bf{S}}}_{{\bf{i}}{\bf{n}}}[\varepsilon ]$$

The system is symmetric about an axis passing through the centre of each optical component. Angular measurements in planes perpendicular to the axis are anticlockwise from horizontal, looking along the axis into the light source. Linear polarization input is defined by the Stokes vector (**S**_**in**_), with an electric field vector orientation *ε°* (angle of polarization measured in degrees). Degree of polarization of the input is defined by the Mueller matrix **M**_**D**,_ with exiting light incident on a radial diattenuator (**M**_**M**_) with orthogonal maximum and minimum principal transmittances *k*_1_*, k*_2_. The output Stokes vector **(S**_**out**_) defines the polarization state of light transmitted through the diattenuator for a radius at angle *θ°*. The model is simplified by assuming that incident light has unit intensity and by ignoring intrinsic ocular retardation (see discussion).

The two-dimensional extent and wavelength-dependence of radial diattenuation is determined by the density function *D*(*r*, *θ, λ*), where *r* is radial distance from the model axis (centre of diattenuator/model macula) at angle *θ°*, and *λ* is the wavelength at which the density function is defined. For the present study it will be assumed that *D* has a maximum value of 1 at 460 nm, the approximate maximum absorption peak of macular pigment.

Using the density function and *S*_0_, the first component of **S**_**out**_ light intensity reaching the photoreceptor outer segments, relative to the intensity incident on the retinal surface, is expressed as a transmittance function (*T*_H_):

2 $${T}_{H}(r,\,\theta ,\,\lambda ,\,{k}_{1},\,{k}_{2},\,P,\,\varepsilon )=1+D(r,\,\theta ,\,\lambda )[(\frac{{k}_{1}+{k}_{2}}{2}-1)+\frac{P({k}_{1}-{k}_{2})\cos \,2(\varepsilon -\theta )}{2}]$$

For unpolarized light, *P* = 0 and Eq. 2 simplifies to the polarization-independent component

3 $${T}_{H}(r,\,\theta ,\,\lambda ,\,{k}_{1},\,{k}_{2})=1+D(r,\,\theta ,\,\lambda )(\frac{{k}_{1}+{k}_{2}}{2}-1)$$

when *D* = 1, this further simplifies to

4 $${T}_{H}({k}_{1},\,{k}_{2})=\frac{{k}_{1}+{k}_{2}}{2}$$

which is the maximum transmittance of the radial diattenuator.

The extent of radial diattenuation is assumed to follow the distribution of human macular pigment^29^, and is defined by a density function consisting of a normalized form of the macular pigment density (MPD) model of Berendschot and van Norren^30^, measured at a wavelength of 460 nm:

5 $$D(r)={A}_{1}{10}^{-{\rho }_{1}r}+{A}_{2}{10}^{-{\rho }_{2}{(r-{x}_{2})}^{2}}$$

where *A*_1,2_, *ρ*_1,2_ and *x*_2_ are parameters that determine the shape of the curve. The function is radially symmetric, and as such is only radius-dependent.

**Figure 1:**
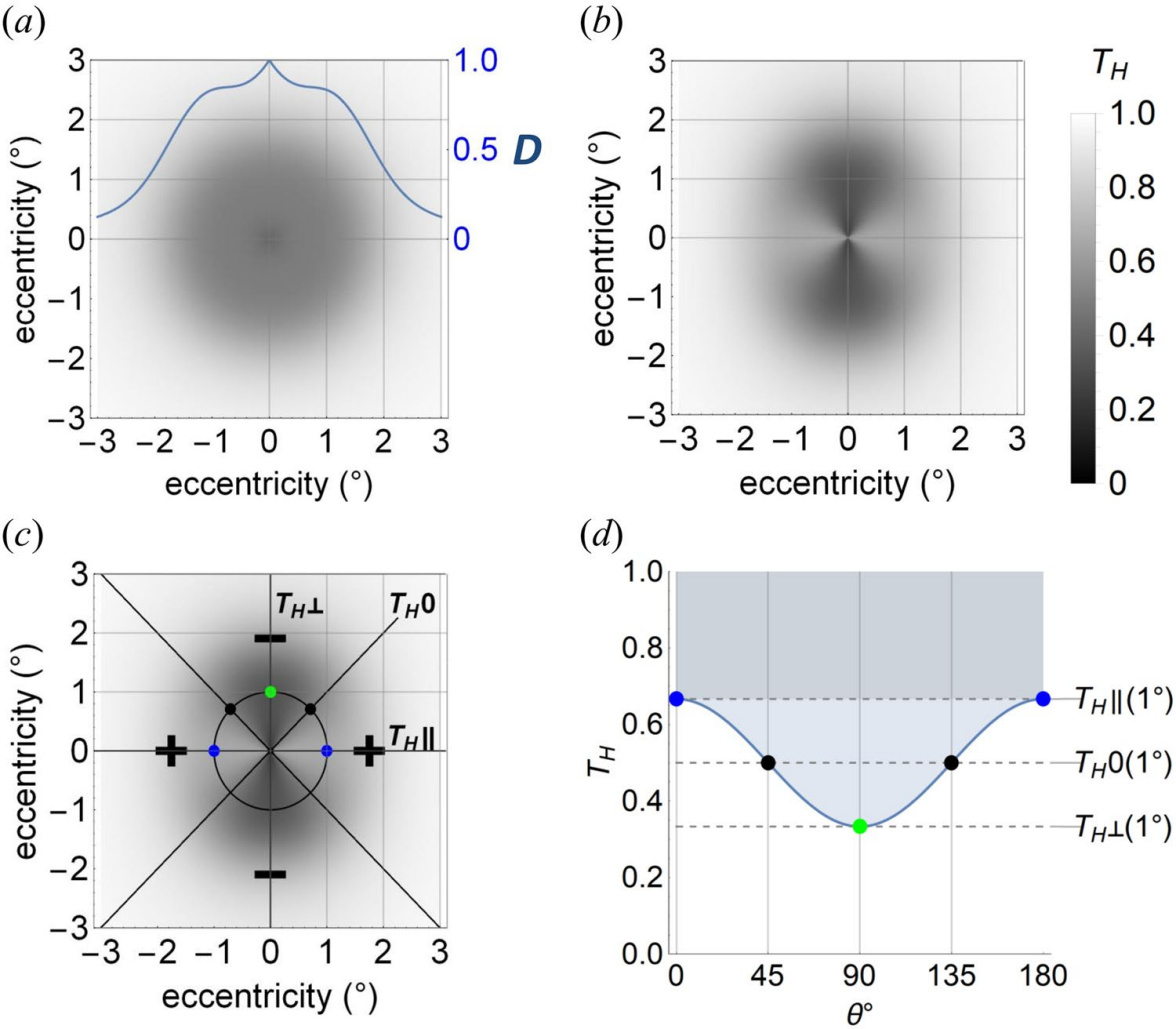
Simulated transmittance of depolarized and linear polarized light incident on a hypothetical radial diattenuator modulated by density function *D*. (**a**) Transmittance of depolarized light for a 6° square field, centred on the diattenuator, with a superimposed cross-sectional plot of *D* (solid blue function). (**b**) As in (**a**) for fully linear polarized light, oriented horizontally. (**c**) Annotated version of (**b**), showing a circle of radius 1° eccentricity (*D* = 0.823) with dots at 0° and 180° (blue), 45° and 135° (black) and 90° (green) from the positive horizontal axis. Horizontal/vertical axes, *T*_*H*_||/*T*_*H*_⊥ are, respectively, parallel and perpendicular to the axis of transmittance of incident polarization. *T*_*H*_ = 0.5 along the ±45° lines, which define quadrants of preferential transmittance (+)/absorption (−). (**d**) Variation of transmittance over half a cycle for the 1° circle defined in (**c**), with corresponding points at 0° (blue), 45° (black), 90° (green), 135° (black), and 180° (blue), plotted as a function of angular measurements (*θ*) in degrees. *T*_*H*_ ||(1°) and *T*_*H*_⊥(1°) indicate parallel and perpendicular transmittance at a radius of 1° eccentricity, respectively. *T*_*H*_ = 0.5 = *T*_*H*_0(1°) at *θ* = 45° and 135° (black dots).

Parameters were chosen to generate a density function equivalent to Sharifzadeh *et al*.’s^31^ Category B (*A*_1_ = 0.25, *A*_2_, = 0.10, *ρ*_1_ = 0.30_,_ *ρ*_2_ = 0.60, *x*_2_ = 1.30). Note that any of Sharifzadeh *et al*.’s categories B–E could have been used (category A has no measurable macular pigment). However, the morphology of category B was chosen because it contains features present in the other categories and is common (22% of individuals). It declines monotonically from a central maximum, plateaus briefly and then diminishes exponentially to a value near zero from 5° eccentricity (Fig. 1a).

The aim of the simulations was to determine the extent of photoreceptor outer segment illumination following transmission through the macular radial diattenuator for light that is either fully depolarized (*P* = 0) or 100% horizontally polarized (*P* = 1, *ε* = 0°). In each case, both hypothetical and experimentally determined physiological values of *k*_1_ and *k*_2_ were used to demonstrate transmission effects.

Estimates of physiological values of *k*_1_ and *k*_2_ were derived from the results of Bone and Landrum^32^, who determined the ratio *G* = *k*_1_/*k*_2_ = 1.1, and the optical density for depolarized light *OD* = −log_10_[(*k*_1_ + *k*_2_)/2] = 0.4. For clarity, the expression uses the notation of the present study. From these values it follows that *k*_1_ = 0.42 and *k*_2_ = 0.38, with a mean value [(*k*_1_ + *k*_2_)/2] of 0.4. Accepting this, photoreceptor outer segments beneath the maximum density of macular pigment receive only 40% of incident depolarized light, while linear polarization increases (decreases) this value by 2% when the polarization axis is parallel (perpendicular) to the radius of the macular partial diattenuator.

### Results and discussion of computational analyses

Whilst the mean transmittance (0.4) is sufficiently large to simulate photoreceptor illumination, the polarization-dependent variation of ±2% does not generate a sufficiently clear graphic demonstration of the polarization effect. For the purposes of generating graphical simulations for print, we used exaggerated, physiologically implausible, principal transmittance values (*k*_1_ = 0.6, *k*_2_ = 0.2) to establish the general properties of light transmission through a radial partial diattenuator, modulated by the density function *D* for both depolarized (*P* = 0) and horizontally linearly polarized (P = 1) light. These computations were then repeated with physiologically plausible principal transmittance values to generate simulations of *in vivo* macular transmission and photoreceptor array illumination.

#### Simulation of photoreceptor array illumination: the effect of polarization with exaggerated principal transmittances (k1 = 0.6, k2 = 0.2)

For depolarized light, the light transmittance simulation (*T*_*H*_0) is radially symmetric and follows Eq. 3 (Fig. 1a). The pattern is different for horizontal linearly polarized light (Fig. 1b,c), in that transmittance is greater than depolarized light along the axis of polarization (*T*_*H*_||) but less than depolarized light along the perpendicular axis (*T*_*H*_⊥). Figure 1d shows the variation in transmittance for horizontally linear polarized light around the upper half of the circle of 1° eccentricity [*T*_*H*_(1°)] shown in Fig. 1c. Transmittance varies sinusoidally, falling from a peak in the horizontal meridian [blue dots, *T*_*H*_|| (1°)] to a mean value corresponding to the transmittance at the same radius for depolarized light [black dots at 45° and 135° from horizontal, *T*_*H*_0(1°)], through to a minimum perpendicular to the axis of polarization [green dot at 90° from horizontal, *T*_*H*_⊥(1°)]. If the central field is divided into quadrants along *T*_*H*_0 (Fig. 1c), there is preferential transmission in quadrants bisected by the plane of polarization and preferential absorption in perpendicular quadrants.

Total transmittance (*T*_*H*_) of linearly polarized light through the radial partial diattenuator can be separated into polarization-dependent and polarization-independent components. The boundary of the polarization-independent component (Fig. 1d, upper, darker shaded rectangular area) is defined by *T*_*H*_|| (*k*_1_ for *D* = 1). The polarization-dependent component of *T*_*H*_ (Fig. 1d, light grey shaded area) varies sinusoidally between *T*_*H*_|| and *T*_*H*_⊥, depending on the angle measured from the plane of linearly polarized light. Amplitude of the polarization-dependent component is *T*_*H*_|| − *T*_*H*_⊥ (and *k*_1_ − *k*_2_ for *D* = 1). Compared with depolarized light, linearly polarized light is preferentially transmitted (absorbed) parallel (perpendicular) to the plane of polarization by (*T*_*H*_|| − *T*_*H*_⊥)/2.

#### Simulation of photoreceptor array illumination: the effect of polarization with physiological principal transmittances (*k*_1_ = 0.42, *k*_2_ = 0.38)

Simulations were repeated with experimentally derived physiological values of *k*_1_ = 0.42, *k*_2_ = 0.38. As the mean of these values is identical to that in the previous simulation set, the spatially-dependent pattern of transmittance for depolarized light (*T*_*H*_0), by Eq. 3, is the same as in Fig. 1a.

**Figure 2:**
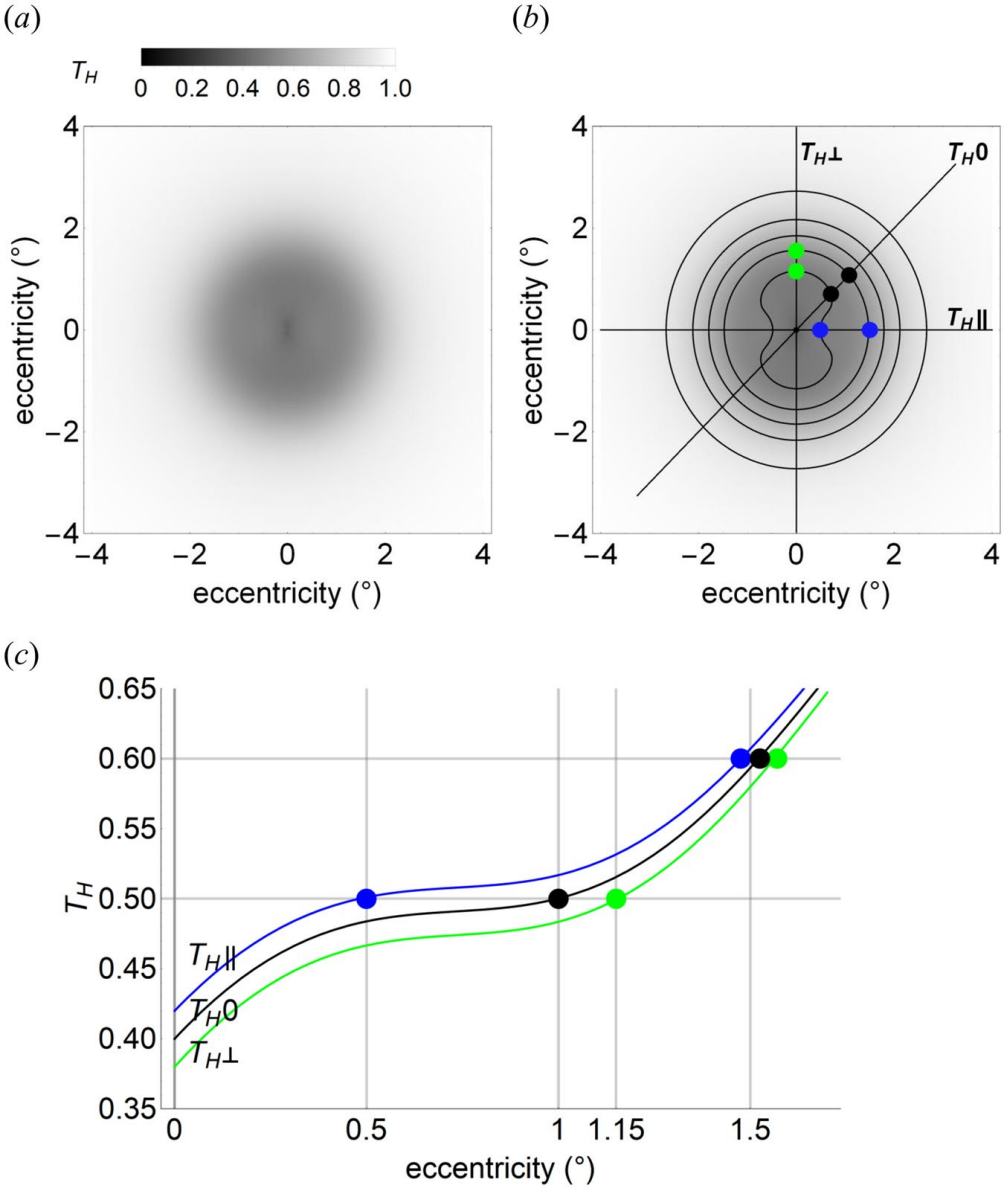
Simulated transmittance of horizontally linear polarized light through a radial diattenuator with physiological partial transmittances *k*_1_ = 0.42, *k*_2_ = 0.38 and density distribution *D*. (**a**) Simulated photoreceptor array illumination for an 8° square field, centred on the diattenuator. (**b**) As in (**a**), annotated with transmittance (*T*_H_) contours, in 0.1 increments, ranging from 0.5 (inner) to 0.9 (outer). The blue/green dots are, respectively, at loci of *T*_*H*_|| = 0.5, 0.6 and *T*_*H*_⊥ = 0.5, 0.6. Axis *T*_*H*_0 indicates orientation (*θ* = 45°, black dots at *T*_*H*_0 = 0.5, 0.6) at which transmittance is equivalent to that for depolarized light. (**c**) The black curve (*T*_*H*_0) shows depolarized light transmittance for eccentricities 0° to 1.7° at any radius (*θ*) of the diattenuator. This curve is also the transmittance of linear horizontal polarized light at a diattenuator radius *θ* = 45°. Blue and green curves are, respectively, transmittances of horizontally polarized light along radii parallel (*θ* = 0°, *T*_*H*_||) and perpendicular (*θ* = 90°, *T*_*H*_⊥) to the polarization axis. Blue and green dots correspond to those in (**b**). The black dot is at eccentricities 1° and 1.52° when transmittance for depolarized light *T*_*H*_0 = 0.5 and 0.6, respectively. Note that *T*_*H*_ > *T*_*H*_0 > *T*_*H*_⊥ for all eccentricities and for all *D* > 0; the eccentricities at which *T*_*H*_|| = *T*_*H*_0 = *T*_*H*_⊥ depend on *D, k*_1_ and *k*_2_ according to Eq. 2

Differences in two-dimensional transmittances for depolarized light (Fig. 1a) and horizontal linearly polarized light (Fig. 2a) are subtle, as the polarization-dependent component of the horizontal linearly polarized light simulation is small compared with the polarization-independent component—this is to be expected from the relative values of *k*_1_ and *k*_2_. Loss of rotational symmetry of the depolarized light transmittance pattern in the horizontally linear polarized light pattern is evident in Fig. 2b, which shows that transmittance contours are either compressed or elongated along *T*_*H*_||or *T*_*H*_⊥ axes, respectively. This is also seen in Fig. 2c, where *T*_*H*_ is plotted against eccentricity along radii parallel (*T*_*H*_||) and perpendicular (*T*_*H*_⊥) to the plane of polarization, and for depolarized light (*T*_*H*_0). Figure 2c also demonstrates that transmittances can be equal [i.e. *T*_*H*_ = *T*_*H*_|| (blue dot) = *T*_*H*_0 (back dot) = *T*_*H*_⊥ (green dot)] at different eccentricities. The points/contours of equal transmittance occur at eccentricities determined by *k*_1_, *k*_2_ and *D*.

#### Simulating MS and HB: the role of neural adaptation

The absorption characteristics and spatial distribution of human macular pigment is such that transmission of a uniform field of either depolarized or linearly polarized light through the macular diattenuator forms a spatially modulated distribution of light on the array of photoreceptors below. Under normal viewing conditions, however, no spatially structured image is perceived. This is so because the image is stabilised on the retina and consequently annulled by adaptational processes^16,33^ similar to those involved with Troxler’s fading^34^.

**Figure 3:**
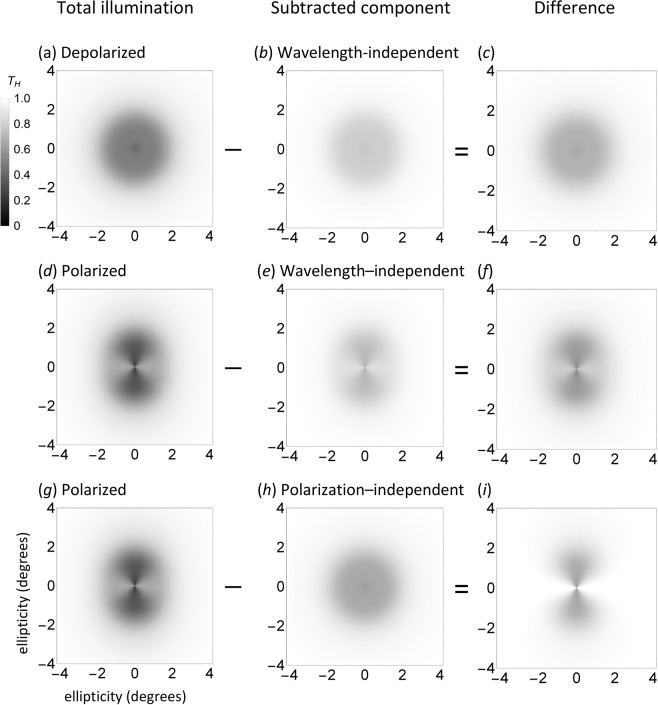
Genesis of MS and HB. Column 1: total photoreceptor array illumination simulation (*k*_1_ = 0.6, *k*_2_ = 0.2, c_λ_ = 1) for (**a**) depolarized light (*P* = 0) and (**d**,**g**) horizontally polarized light (*P* = 1, *ε* = 0). Column 2: Unchanging (adapted/subtracted) component that is (**b**,**e**) wavelength-independent (*k*_1_ = 0.6, *k*_2_ = 0.2, c_λ_ = 0.4) or (**h**) polarization-independent (*k*_1_ = *k*_2_ = 0.6, c_λ_ = 1). Column 3: difference between total transmission and unchanging components, simulating (**c**) MS with depolarized light (*P* = 0, *k*_1_ = 0.6, *k*_2_ = 0.2, c_λ_ = 1 − 0.4 = 0.6), (**f**) MS with horizontally linear polarized light (as (**c**) but *P* = 1) and (**f**) HB (*P* = 1, *k*_1_ = 1, *k*_2_ = 0.6, c_λ_ = 1). Axes and scales as in Fig. 2. See text for parameters and other details.

MS is perceived when viewing a uniformly illuminated field of unpolarized light whose wavelength is alternated between one that is predominantly absorbed and one that is predominantly transmitted by macular pigment. HB is perceived when viewing a uniformly illuminated field of linear polarized light in which the **E**-vector is constantly changing orientation (e.g. alternating through a set angular distance or rotating) and whose wavelength is predominantly absorbed by macular pigment. Transmission of the alternating viewing states through the diattenuator generates photoreceptor array illumination consisting of two superimposed spatially patterned components. The two components are either common to the alternate viewing states or not (i.e. with each phenomenon, both changing and unchanging distributions of patterned light fall on the photoreceptor array). We assume that the unchanging patterned image is negated in the same manner as spatially stabilised retinal images, namely, through the process of neural adaptation. Here, we model this adaptational process by subtracting the unchanging component of illumination from the total photoreceptor array illumination. The results of these computational analyses with exaggerated principal transmittances are shown in Fig. 3.

For MS, the unchanging component is the proportion of light absorbed common to both viewing wavelengths. In the present demonstration, the constant components (Fig. 3b,e) are the total illuminations (Fig. 3a,d) attenuated by a wavelength dependent factor *c*_λ_, arbitrarily given a value of 0.4. The value of *c*_λ_ could approach zero if there is total transmission (i.e. no absorption) generated by *D* at the chosen wavelength (e.g. there is minimal absorption of macular pigment for red wavelengths). MS simulations are generated for alternating wavelengths (*c*_λ_ = 1 or 0.4) that are depolarized (Fig. 3a–c) or polarized (Fig. 3d–f). MS elicited with depolarized light (Fig. 3c) follows the spatial distribution of the density function (Fig. 1a). MS elicited with horizontally linearly polarized light (Fig. 3f) also follows the density function, but is lighter along the axis of polarization and darker orthogonal to this axis. This morphology implies that, *in vivo*, MS observed with polarized light will have a smaller (larger) diameter parallel (perpendicular) to the **E**-vector orientation compared with the image generated with depolarized light.

For HB, the unchanging component (Fig. 3h) is the proportion of light that is common to axes that are perpendicular and parallel to the incident **E**-vector (i.e. the polarization-independent component; see Fig. 1d). The effect of subtracting this component from the total illumination (Fig. 3g) is to equalise the perceived illumination along the **E**-vector axis to that of the background, yielding the characteristic pattern of HB (Fig. 3i).

## Experimental

Two experimentally testable predictions arise from the simulations derived from our computational model:

1. **The perceived size of MS is polarization-dependent**. In particular, its diameter is dependent on incident **E**-vector orientation and the degree of polarization. The model expressed in Eq. 2 predicts that the diameter of MS will vary with degree of polarization and **E**-vector angle when the degree of polarization is greater than zero, such that the diameter of MS in the horizontal plane when the light is vertically polarized (dMS ⊥) will be greater than the diameter of MS when the light is completely depolarised (dMS0), which is greater still than the diameter of MS when the light is horizontally polarized (dMS ||).
2. **The perceived sizes of MS and HB will be correlated**.

The predictions were tested experimentally *in vivo* in human participants by determining the horizontal diameter of MS when observed with depolarized light, horizontally linear polarized light and vertically linear polarized light. For comparison, the dimensions of HB were measured under similar conditions of polarization.

### Apparatus and experimental method

**Figure 4:**
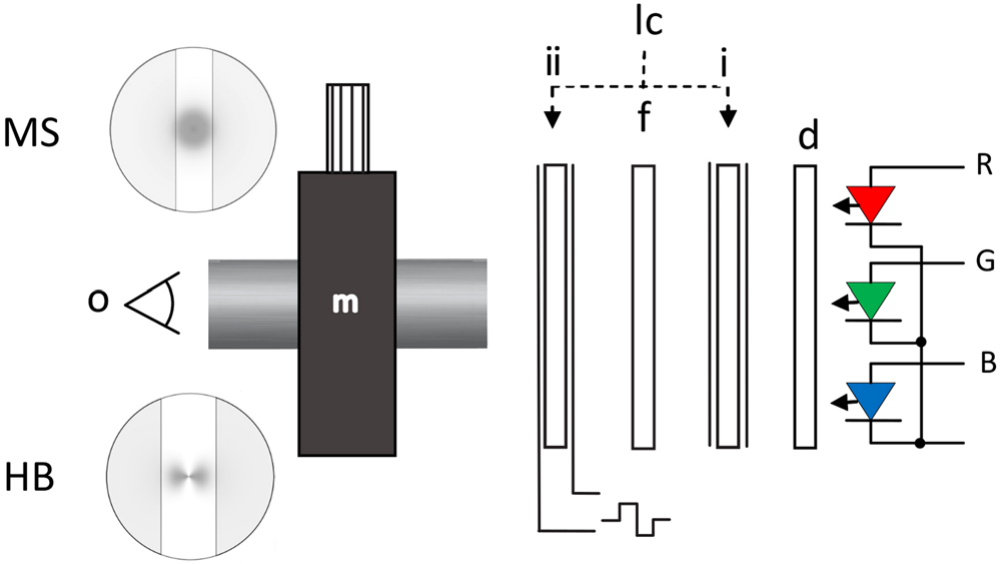
Schematic of experimental setup (not to scale). Red (R), Green (G) and Blue (B) LED light source; d, diffuser/depolarizer; lc liquid crystal polarization rotator in position (i) or (ii); f, filter tray; m, filar micrometer eyepiece. MS and HB: monochrome simulations of typical Maxwell’s spot and Haidinger’s brushes percepts between callipers (vertical lines/shaded areas) set to the image boundary.

The apparatus (Fig. 4) comprised a controllable tri-colour LED light source (R,G,B), a diffuser/depolarizer, filter rack and a filar micrometer eyepiece (Malies Instruments Ltd. UK. #5386) calibrated in degrees of visual angle subtended on the visual axis at the surface of the eye. A liquid crystal polarization rotator was placed either behind the filter rack (position i; neutral) or between the filter rack and eyepiece (position ii; polarization rotating). The polarization rotator was a single element twisted nematic LCD ‘light shutter’ (Adafruit Industries, product ID 3627), from which the two polarizing filters had been removed. The filter rack had three settings: settings 1 and 2 were linear polarizing filters with axes orientated either vertically (setting 1) or horizontally (setting 2) when moved into position. Setting 3 was an aperture stop that transmitted depolarized light to the eye, limited to the same intensity as settings 1 and 2.

Intensity, spectral and polarization characteristics of light exiting the eyepiece were measured using a polarimeter, comprising a spectrometer (USB2000 Ocean Optics USA.), Glan-Thompson polarizer and a Fresnel rhomb achromatic quarter-wave retarder^35^. Peak spectral output was R = 633 nm, G = 519 nm and B = 456 nm. For the purple setting, R:G:B = 0.37:0.00:1.00; for the orange setting R:G:B = 1.00:0.76:0.00 (where a value of 1 is maximum intensity for that channel). Light from the diffuser (and for setting 3) was fully depolarized.

For MS viewing, the polarization rotator was in a neutral position (i in Fig. 4), and the light source was alternated between purple and orange at a rate of 1 Hz, the frequency at which MS appeared most salient for this setup. MS was observed as pink/purple rings when viewed against the purple background, or as a complementary afterimage when viewed against the orange background. The observer’s task was to set the micrometer callipers to the horizontal width of the perceived MS for each of settings 1–3, which were presented in random order. The measurements were designated dMS || for setting 1, dMS ⊥ for setting 2 and dMS0 for setting 3. Results were averaged from three trials for each setting. Additionally, observers were asked to describe the percept and any differences between images generated by different settings.

For HB viewing, the light source was constant purple. The polarization rotator was in position ii and the polarizer set horizontally (setting 1). The polarization rotator was activated by external circuitry to alternate the state of polarization between horizontal and vertical at a rate of 2 Hz, the frequency at which HB was most salient for this setup. HB was observed as pink/purple brushes alternating between horizontal and vertical against the purple background. The observer’s task was to set the micrometer callipers to the perceived horizontal width of HB (i.e. when the incident polarization was vertically orientated, equivalent to setting 2). Results were averaged from three trials. Control tests were performed with the activated polarization rotator and setting 3 (i.e. with depolarized illumination). As before, observers were asked to describe the percept and any differences from previous settings.

Ten participants (7 male, 3 female) aged between 18 and 62 yrs were tested. All individuals had normal or corrected-to-normal visual acuity, no evidence of eye disease and no history, or family history, of eye disease. All participants gave informed consent, and testing was in accordance with the relevant guidelines and regulations of the tenets of the Declaration of Helsinki. The Aston University Ethics Committee approved all experimental protocols.

### Experimental results

For each of settings 1–3, all observers reported the appearance of MS to be markedly different from that of HB. Although variations in the MS pattern between settings were noted by observers, in no case was the generated pattern thought to resemble HB.

**Figure 5:**
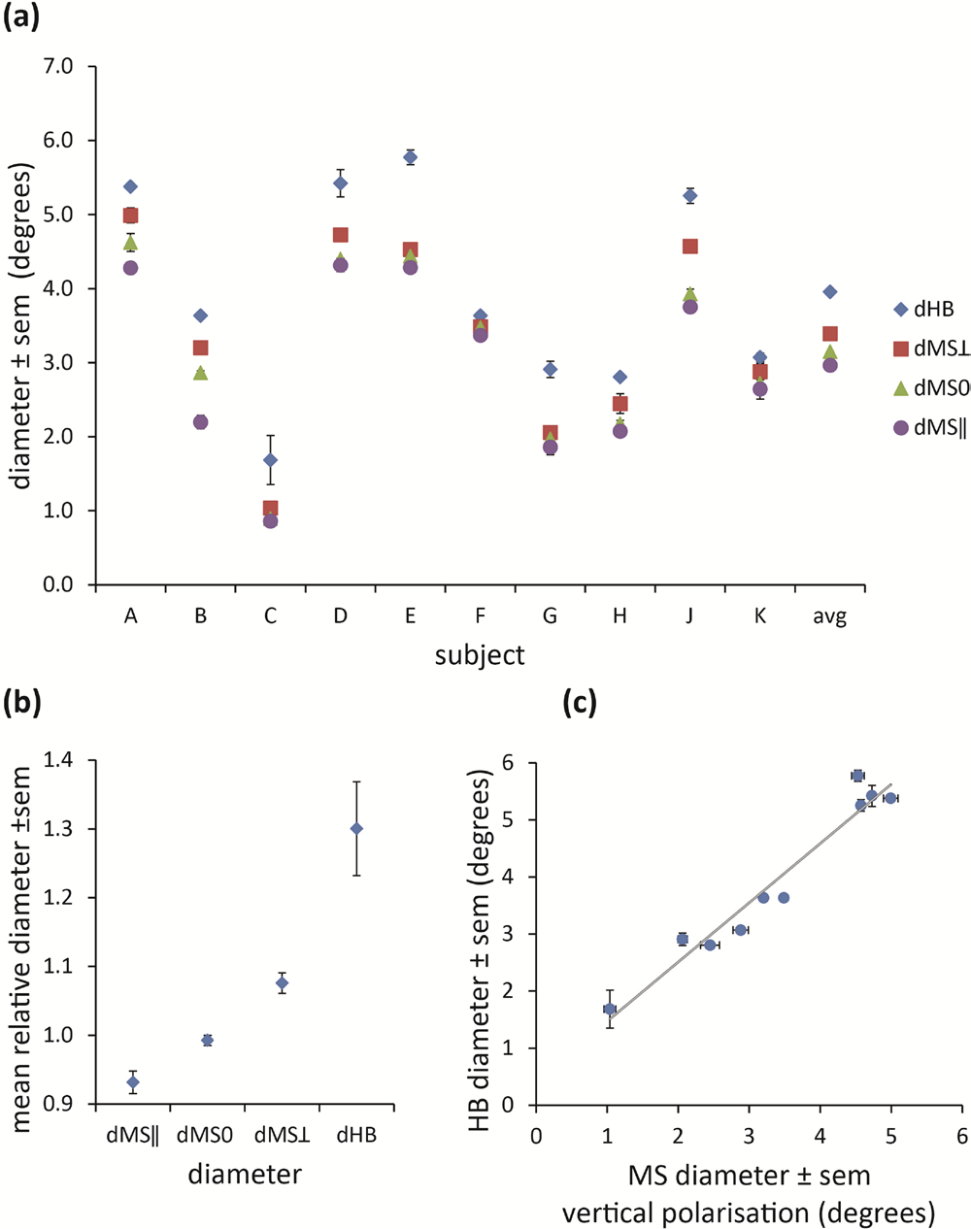
Experimental results. (**a**) Measured horizontal diameters for HB and MS for the ten observers. See text for details. (**b**) Measured diameters expressed as a fraction of the mean Maxwell’s spot values, averaged across all observers. (**c**) Correlation between MS diameter (vertical polarization, dMS ⊥) and HB diameter for each individual. Pearson product correlation coefficient *r* = 0.97 (*p* ≪ 0.01) and regression equation dHB = 1.04 × dMSmean +0.43. The vertical and horizontal error bars show ± 1 SEM.

The measured horizontal diameter data are presented in Fig. 5 and the full data set is given in the Supplementary Dataset. Whilst there was considerable inter-subject variation in the measured values (Fig. 5a), relative values for each diameter, expressed as a fraction of the mean of all three MS measurements [dMSmean ≈ (dMS || + dMS0 + dMS ⊥)/3], were similar for each observer (Fig. 5b) and in agreement with theoretical predictions (Fig. 2). Note also that the diameter of HB is well correlated with the dMS ⊥ (*r*^2^ = 0.95, *p* ≪ 0.01, see Fig. 5c), where both phenomena are observed with vertically polarized light. There were similar correlations between dHB and the three other MS diameters (*r*^2^ = 0.94, *p* ≪ 0.01, for dHB v MS mean; *r*^2^ = 0.92, *p* ≪ 0.01, for dHB v dMS ||; *r*^2^ = 0.94, *p* ≪ 0.01, for dHB v dMS0).

When configured for HB viewing (constant purple illumination with active polarization rotator), neither HB nor MS were perceived with fully depolarized light (setting 3). HB oscillating at 2 Hz was perceived continuously for both polarization settings (settings 1 and 2), but faded within approximately 3 seconds when the polarization rotator was inactive or removed.

## Discussion and Conclusions

The phenomena of Maxwell’s Spot (MS) has been linked to that of Haidinger’s Brushes (HB) since its first description^13^, despite the manifest differences in their morphology and mode of generation. The accepted view is that both phenomena are dependent on the unique geometric distribution of macular pigment within the human retina^31,36^. Using a combined theoretical and *in vivo* experimental approach, we have clarified the relationship between MS and HB, and detailed the important role played by adaptive mechanisms in their genesis. In particular, our results provide evidence that both MS and HB are consequent upon the degree of polarization-dependent differential absorption by a wavelength-dependent, radially arranged macular diattenuator. Establishing the interrelationship between these phenomena advances our understanding of their psychophysical basis and aids their potential clinical utility.

The theoretical part of this study used an established radial diattenuator model to simulate macular photoreceptor array illumination for both hypothetical and physiologically relevant macular pigment principal transmittances and spatial densities. Although the general model (expressed in Eq. 2) is applicable to any polarization state, only depolarized and linearly polarized light are considered here, as these are the states used to generate MS and HB. We did not consider the effects of corneal retardation as it has no effect on MS observed with depolarized light, and only becomes significant for HB in a small number of individuals with high corneal retardations^27,37–39^.

The normalised distribution of macular pigment was used as a proxy for the density function that determines the two-dimensional distribution of macular diattenuation. Whilst alternative non-macular pigment based mechanisms have been proposed for the generation of both MS^40^ and HB (e.g. LeFloch *et al*. 2012), they were not considered here because of the overwhelming experimental support in favour of macular pigment within Henle’s layer being the basis for the generation of MS and the site of diattenuation necessary for the generation of HB^18^.

The simulated photoreceptor illumination pattern depends on polarization state, being circularly symmetric for depolarized light and having a two-fold rotational symmetry for linear polarized light, with the greatest (least) density parallel (perpendicular) to **E**-vector orientation. Paired simulations of photoreceptor array illumination were generated to represent the alternate viewing states for the generation of each phenomenon. Simulation of each phenomenon was generated by subtracting those components of photoreceptor array illumination common to both alternate states (Fig. 3). We propose that the neurosensory equivalent of the computational subtraction is negation of stabilised retinal images by adaptational processes. The effect is demonstrated *in vivo* in Supplementary Animation S1. The animation alternates between two images based on Fig. 2(a) simulating photoreceptor array illumination for horizontal and vertically polarized light: maintaining central fixation results in Troxler fading of the constant component with preservation of the alternating polarization-dependent component thereby generating a HB-like percept on an apparently uniform background.

The experimental part of the study measured and compared the angular subtense (measured as diameter) of MS and HB under appropriate viewing conditions (namely, constant state of polarization with alternating wavelengths for MS; constant wavelength with alternating polarization state for HB). The horizontal diameter of MS when observed by subjects under depolarized light (dMS0, mean ± SEM = 3.1° ± 0.4°) was consistent with previous measurements, which range from 1.25° to 4.5° diameter^14,40^. The variability in measurements shown in Fig. 5a is to be expected, given the known variability in density and spatial distribution of macular pigments between individuals^31,41^. The observed diameter of HB (dHB, mean ± SEM = 4.0° ± 0.4°) is also comparable with previous reports of approximately 5° diameter^18,22^.

Whilst our model simulates the known form and dimensions of MS and HB, it also predicts a previously undescribed MS-like percept, which we term polarization-modified MS (pMS) patterns. Such patterns are formed using linearly polarized light, but under alternate wavelength viewing states that favour the perception of MS (Fig. 3d–f). While pMS resembles the classic MS pattern, its morphology is amplified (attenuated) along the direction orthogonal (parallel) to the axis of polarization. The existence of pMS patterns, and hence the validity of the theoretical model, is apparent from the experimental results (Fig. 5) that show an expected decrease (increase) in horizontal diameter of the entoptic phenomenon observed with horizontally (vertically) polarized light. Furthermore, in accordance with theoretical predictions, the mean of the diameters measured approaches that for MS observed with depolarized light. The experimental data also support a further prediction of the model, that the measured diameter of HB correlates with that of MS (Fig. 5c). The latter reinforces the hypothesis that both phenomena have a common origin.

The measured HB diameters were approximately 30% greater than the MS counterpart (Fig. 5b,c). This was an unexpected finding, given that the attenuation of the polarization-independent component is an order of magnitude greater than the maximum amplitude of the polarization-dependent component. The reasons for this remain an open question and are currently being investigated, but likely relate to the different experimental conditions of illumination and different states of adaptation required to observe each phenomenon.

The macular dependence and interrelationship between HB, MS and pMS established in this study suggest that they can all be used as tests of macular function in health and disease. Such tests are well documented for HB^20–22,25,26^, but the clinical utility of MS and pMS deserves further investigation, particularly with respect to the diagnosis/assessment of macular disease such as age-related macular degeneration and diabetic maculopathy.

In his original report^13^, Maxwell wrote ‘*the brushes of Haidinger are well seen in connexion with the spot*’. This statement is supported by both theoretical argument and *in vivo* measures detailed in the present study. Maxwell’s further, as yet unchallenged, conjecture reads ‘… *and the fact of the brushes being the spot analysed by polarized light becomes evident*’. This statement is not supported by the present study. Our theoretical arguments, computational simulations and *in vivo* measures reveal that MS generated using polarized light remains a spot, albeit one modified in appearance when compared with MS generated with depolarized light. With polarized light, the shadow of macular pigment on the photoreceptor array is perceived as HB only if the conditions of observation favour adaptive negation of the transmitted polarization-independent component.

## Supplementary information

Animation.

Supplementary Dataset 1.
