# Supplementary figures and images for: Polarization perception in humans: on the origin of and relationship between Maxwell’s spot and Haidinger’s brushes
Source: Sci Rep. 2020 Jan 10;10:108. doi: 10.1038/s41598-019-56916-8 (PMC6954220; doi:10.1038/s41598-019-56916-8)

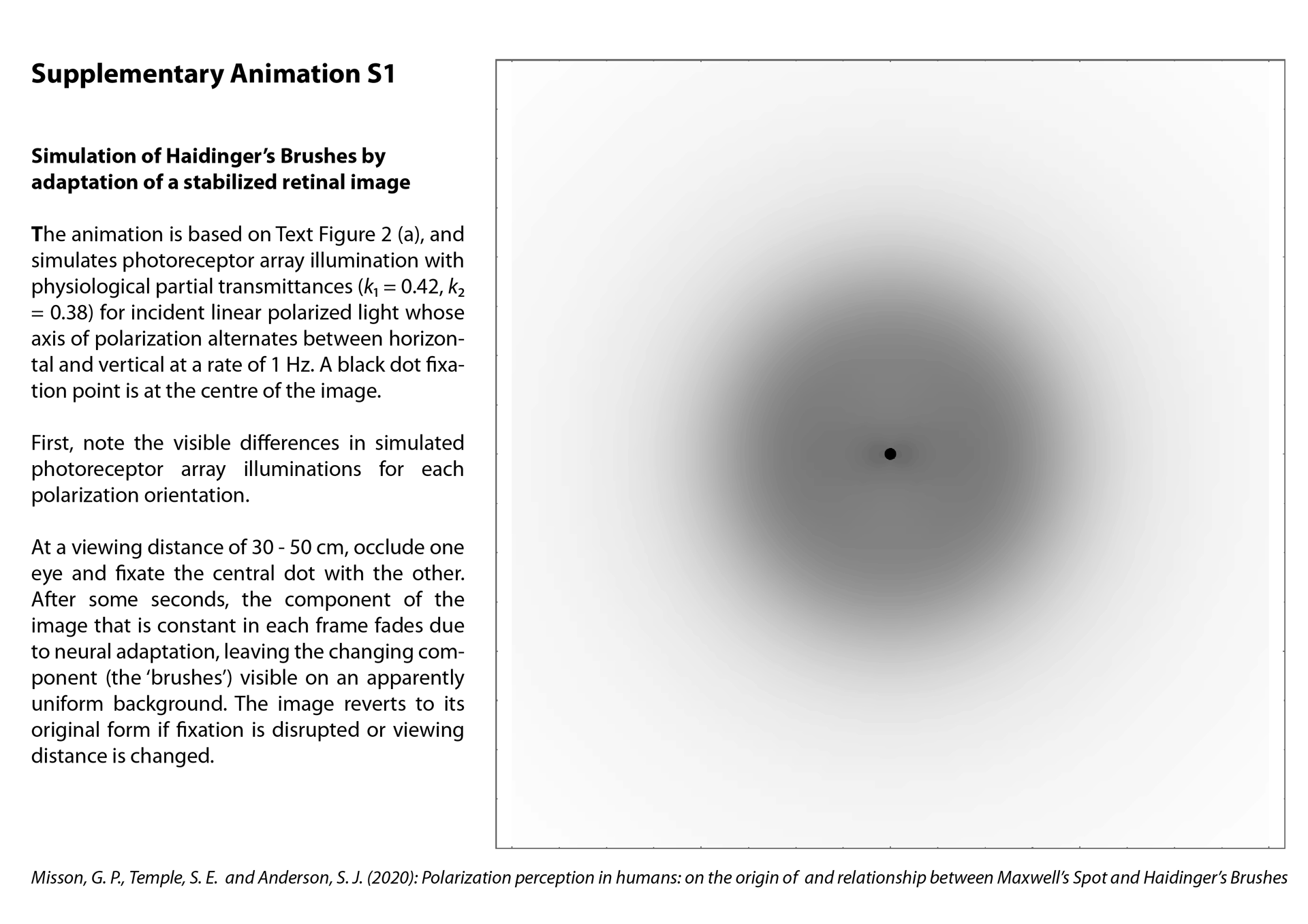

Supplement: Supplementary file 1 — Animation. [file 41598_2019_56916_MOESM1_ESM.gif]
